# Correction: Petry-Schmelzer et al. Selecting the Most Effective DBS Contact in Essential Tremor Patients Based on Individual Tractography. *Brain Sci*. 2020, *10*, 1015

**DOI:** 10.3390/brainsci12010064

**Published:** 2021-12-31

**Authors:** Jan Niklas Petry-Schmelzer, Till A. Dembek, Julia K. Steffen, Hannah Jergas, Haidar S. Dafsari, Gereon R. Fink, Veerle Visser-Vandewalle, Michael T. Barbe

**Affiliations:** 1 Department of Neurology, Faculty of Medicine and University Hospital Cologne, University of Cologne, 50923 Köln, Germany; jan.petry-schmelzer@uk-koeln.de (J.N.P.-S.); julia.steffen@uk-koeln.de (J.K.S.); hannah.jergas@uk-koeln.de (H.J.); haidar.dafsari@uk-koeln.de (H.S.D.); gereon.fink@uk-koeln.de (G.R.F.); michael.barbe@uk-koeln.de (M.T.B.); 2 Cognitive Neuroscience, Institute of Neuroscience and Medicine (INM-3), Research Center Jülich, 52428 Jülich, Germany; 3 Department of Stereotactic and Functional Neurosurgery, Institute of Medicine and University Hospital Cologne, University of Cologne, 50923 Köln, Germany; veerle.visser-vandewalle@uk-koeln.de

The authors wish to make the following correction to [1]. There was a mistake in analyzing the ranking of the contacts, as depicted in Figure 1 of the original manuscript. However, the main findings remain unchanged and numerically even improve, as the outlier (original Figure 1, Patient 5, right hemisphere) was only due to the mistake in contact ranking (see corrected Figure 1).

In detail, the reported results section and the referring numeric results throughout the manuscript need to be corrected as follows:

“In all (previously 92.9% of) investigated DBS leads, the contact with the best clinical effect was the contact with the highest or second-highest DRTT overlap.”

“In 64.3% (previously 71.4%) of the cases (9 of 14 hemispheres (previously 10 of 14)), the contact with the highest overlap with the individual DRTT showed the best clinical outcome or was among those with the best outcome if more than one contact showed equal tremor improvement. In 5 of 5 (previously 3 of 4) of the remaining hemispheres, the contact with the second-highest overlap with the individual DRTT showed the best clinical outcome. When only investigating directional contacts, in 57.1% (previously 64.3%) of the cases, the directional contact with the highest DRTT-overlap also had the best tremor improvement.”

This correction also partly extends to the discussion:

“The overlap with the DRTT determined one of the most effective contacts in 64.3% (previously 71.4%). When also considering the contact with the second-highest overlap, this increased to 100% (previously 92.9%) of cases. In other words, if one had only interrogated the two contacts with the highest DRTT-overlap, a contact with an optimal outcome would have been determined in each (previously 13/14) hemisphere.”

“In the remaining hemisphere (patient 5, right hemisphere), in which the most effective contact ranked worse, i.e., in seventh place, the contact with the highest overlap still was on the same directional level as the most effective contact and improved tremor by 75%.” This sentence is deleted.

“Only considering directional contacts, the chance of activating the most effective directional contact without clinical testing increases from 16.7% (1 out of 6) to 57.1% (previously 64.3%) when using the in silico approach presented here.”

Note that the resulting conclusions remain untouched by this corrigendum. The authors would like to apologize for any inconveniences caused by these changes.

## Figures and Tables

**Figure 1:**
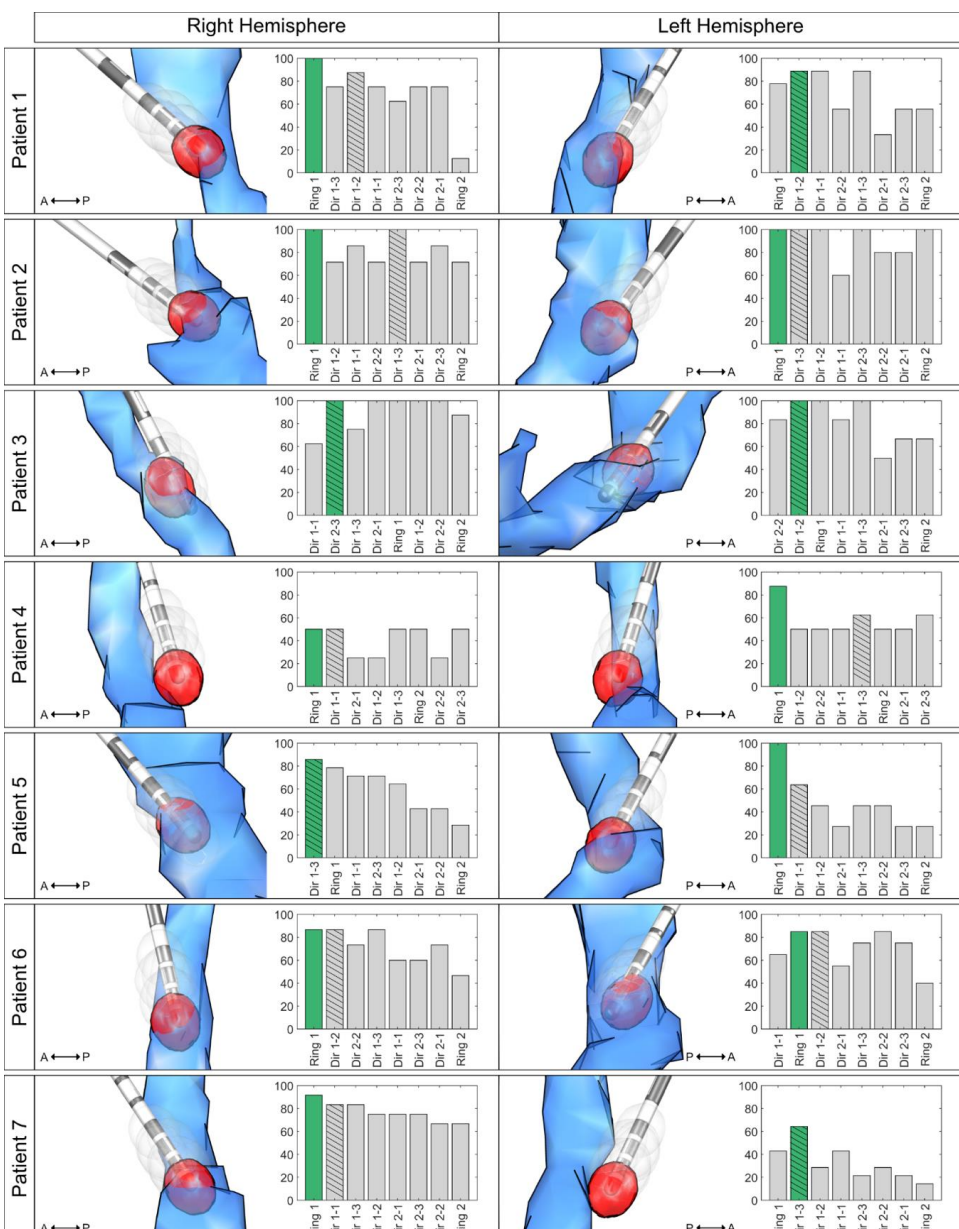
Contact Ranking. Bar plots illustrate the ranking of the overlap, with the individual dentatorubrothalamic tract (DRTT) per contact on the x axis (highest overlap to lowest overlap) and the improvement in tremor control in % on the y axis. The most effective contact is marked in green (with the highest overlap in cases where more than one contact had the best improvement), and the most effective directional contact bar is hatched. The respective left column illustrates the relation between the generated volumes of tissue activated (VTAs, gray) and the DRTT (blue) and the respective lead in the medial view. The VTA with the highest DRTT overlap is highlighted in red. For illustration, only the 10% highest values of the track probability map are shown. Abbreviations: A = anterior; DRTT = dentatorubrothalamic tract; P = posterior; VTA = volume of tissue activated; Dir 1 = ventral directional level; Dir 2 = dorsal directional level.
